# Supplementary material for: A patient-centered comparative effectiveness research study of culturally appropriate options for diabetes self-management
Source: medRxiv. 2023 Feb 8:2023.01.31.23285236. Preprint. [Version 2] doi: 10.1101/2023.01.31.23285236 (PMC9915824; doi:10.1101/2023.01.31.23285236)
Supplement: 1 [file NIHPP2023.01.31.23285236v2-supplement-1.pdf]

medRxiv preprint doi: https://doi.org/10.1101/2023.01.31.23285236; this version posted February 8, 2023. The copyright holder for this preprint (which was not certified by peer review) is the author/funder, who has granted medRxiv a license to display the preprint in perpetuity.
It is made available under a CC-BY 4.0 International license .

Supplement

Additional questions for Patients

Table S 1. Two added questions to the but were not included in the total scoring.

11. La persona que me brinda apoyo social es parte importante en el manejo de mi condición. / My social support is an important part of managing my condition.

    1 Totalmente de acuerdo / Strongly agree

    2 Un poco de acuerdo / Somewhat agree

    3 Neutral / Neutral

    4 Un poco en desacuerdo / Somewhat disagree

    5 Totalmente en desacuerdo / Strongly disagree

12. [prog] me provee herramientas y recursos para manejar mejor mi condición. / [prog] provides me with tools and resources to better manage my condition.

    1 Totalmente de acuerdo / Strongly agree

    2 Un poco de acuerdo / Somewhat agree

    3 Neutral / Neutral

    4 Un poco en desacuerdo / Somewhat disagree

    5 Totalmente en desacuerdo / Strongly disagree

medRxiv preprint doi: https://doi.org/10.1101/2023.01.31.23285236; this version posted February 8, 2023. The copyright holder for this preprint (which was not certified by peer review) is the author/funder, who has granted medRxiv a license to display the preprint in perpetuity.
It is made available under a CC-BY 4.0 International license .

Diabetes Knowledge Questionnaire

Table S 2. Patients incorrectly answered "yes" to these DKQ questions most often. Interestingly, 100% of all patients correctly answered the question whether people with diabetes should take extra care when cutting their toenails.

**Incorrect**	**Number**	**Question**
86.2%	Question #17	*"A person with diabetes should cleanse a cut with iodine and alcohol"*
80.1%	Question #1	*"Eating too much sugar and other sweet foods is a cause of diabetes"*
79.8%	Additional Question #25	*"Drinking too many sugary drinks, such as sodas, is a cause of diabetes"*
75.3%	Question #21	*"Shaking and sweating are signs of high blood sugar"*
66.0%	Question #24	*"A diet for people with diabetes consists mostly of special foods"*
54.1%	Question #3	*"Diabetes is caused by failure of the kidneys to keep sugar out of the urine"*
51.7%	Question #12	*"An insulin reaction is caused by too much food"*
44.3%	Question #22	*"Frequent urination and thirst are signs of low blood sugar"*
25.5%	Question #13	*"Medication is more important than diet and exercise to control my diabetes"*

medRxiv preprint doi: https://doi.org/10.1101/2023.01.31.23285236; this version posted February 8, 2023. The copyright holder for this preprint (which was not certified by peer review) is the author/funder, who has granted medRxiv a license to display the preprint in perpetuity.
It is made available under a CC-BY 4.0 International license .

Patient Activation Measure

Table S 3. Patients answered "disagree" or "neutral" to these PAM questions most often (questions are paraphrased so as to conform to the PAM license agreement).

**Disagree or Neutral**	**Number**	**Question**
20.5%	Question #4	Confident about choosing medical attention or self-care for a health issue.
26.9%	Question #7	Can stick to positive diet and exercise goals.
20.4%	Question #8	Knowledgeable in preventing health issues.
16.8%	Question #9	Confident in my ability to find solutions to new health problems.
19.9%	Question #10	Even during stress, can stick to positive diet and exercise goals.
